# Rehabilitation use in multiple sclerosis: Do illness representations matter?

**DOI:** 10.1002/brb3.953

**Published:** 2018-04-24

**Authors:** Manuela Glattacker, Jürgen M. Giesler, Katharina Klindtworth, Angelika Nebe

**Affiliations:** ^1^ Section of Health Care Research and Rehabilitation Research Medical Center—University of Freiburg Faculty of Medicine University of Freiburg Freiburg Germany; ^2^ German Pension Insurance Berlin Germany

**Keywords:** illness representation, multiple sclerosis, rehabilitation, utilization

## Abstract

**Objectives:**

Multidisciplinary rehabilitation improves illness outcomes and is recommended in clinical guidelines for multiple sclerosis (MS). However, many people with MS do not make use of rehabilitation. We do not know much about the barriers to the use of rehabilitation in MS, but in other patient groups, illness representations have proven to be predictors of service utilization. Therefore, the aim of our study was to explore whether, in patients with MS, illness representations are associated with self‐reports of rehabilitation use in the past and the intention to use rehabilitation in the future, beyond sociodemographic and illness‐related factors.

**Materials and Methods:**

Patients were recruited in a cross‐sectional nationwide online survey in Germany. Hierarchical binary logistic regression analysis was used to analyze whether illness representations are associated with the use of rehabilitation in the past and the intention to use rehabilitation in the future, over and above socio‐demographic and illness‐related variables.

**Results:**

There were 590 patients, who had MS, participating in the study. Illness representations were correlated to both outcome variables beyond sociodemographic and illness‐related factors: The probabilities of having the intention to use rehabilitation and of making using of rehabilitation were higher in patients who believed that their MS was controllable by treatment and perceived that their MS would have severe consequences.

**Conclusions:**

Our data suggest that addressing patients’ illness representations may facilitate the intention to use and the use of multimodal rehabilitation, contributing to better illness outcomes.

## 1 INTRODUCTION

Multiple Sclerosis (MS) is an autoimmune neurological disease which affects approximately 2.3 million people worldwide (Multiple Sclerosis International Federation, 2013). At the time of diagnosis, 85% of patients have a relapsing‐remitting form of MS, and within this subgroup of patients, around 80% will develop secondary progressive MS (Multiple Sclerosis International Federation, 2013). MS is characterized by a high inter‐ and intraindividual variability (Beer, Khan, & Kesselring, 2012), involving various combinations of physical, cognitive, psychosocial, behavioral, and environmental problems (Khan, Turner‐Stokes, Ng, Kilpatrick, & Amatya, 2007). These include impairments related to strength, coordination and vision, spasticity, cognitive deficits, bowel and bladder problems, sexual dysfunctions, pain, and fatigue, which result in limiting activity, for example in mobility and self‐care, and in restricting participation in society (Beer et al., 2012; Khan & Amatya, 2017; Khan et al., 2007; Multiple Sclerosis International Federation, 2013). In light of this complexity, there is—besides pharmacological treatment—a need for comprehensive multidisciplinary medical rehabilitation (Beer et al., 2012; Khan & Amatya, 2017; Rasova et al., 2010), which is also recommended in clinical guidelines for MS (DGN, 2014; NICE, 2014). Rehabilitation realizes patient centredness, uses a biopsychosocial approach, and is functionally oriented with the aim of maximizing activity and participation (Beer et al., 2012; Khan et al., 2007). To achieve these goals, comprehensive rehabilitation programs include a large body of functional interventions, especially physical treatment methods, occupational, speech, and swallowing therapy, as well as psychological interventions (Beer et al., 2012; Rasova et al., 2010). In Germany, multimodal, multiprofessional MS rehabilitation is mostly provided in an inpatient setting. It lasts from 3 to 6 weeks, and the patient has generally multiple therapy sessions a day on workdays. Despite a lack of high‐quality evidence for many rehabilitative treatments seen individually (Khan & Amatya, 2017), evidence for multidisciplinary rehabilitation in terms of improvements in activity and participation is supported (Khan & Amatya, 2017; Khan et al., 2007). Nevertheless, as in other European countries (Helland, Holmøy, & Gulbrandsen, 2015), many people in Germany with MS do not make use of rehabilitation (Nebe & Naumann, 2015), although multidisciplinary rehabilitation is widely available for these patients.

Not much is known about the barriers to the use of rehabilitation in MS. However, as Helland et al. (2015) showed in their qualitative study about the barriers and facilitators related to rehabilitation stays in MS, patients’ assumptions and expectations about rehabilitation were important factors influencing the use of MS rehabilitation. Covering patients’ assumptions about illness (relating to its symptoms, cause, timeline, control/cure, and consequences (Petrie & Weinman, 2006)), the patients’ illness representations, which are theoretically embedded in the well‐evaluated Common Sense Model of self‐regulation (CSM; (Leventhal, Leventhal, & Cameron, 2001)), have initiated a large body of research. Consistent with the assumptions of the CSM, illness representations have proven to be important predictors of various aspects of self‐regulation, such as adherence, adjustment, and work participation in many acute and chronic illnesses (Hagger & Orbell, 2003; Hoving, van der Meer, Volkova, & Frings‐Dresen, 2010; Petrie & Weinman, 2006). Furthermore, in the context of cardiac rehabilitation, illness representations turned out to be predictors of attendance at rehabilitation (French, Cooper, & Weinman, 2006; Whitmarsh, Koutantji, & Sidell, 2003) and dropout from outpatient rehabilitation (Yohannes, Yalfani, Doherty, & Bundy, 2007).

Studies about illness representations in populations with MS are surprisingly scarce (Dennison, Moss‐Morris, & Chalder, 2009; Dennsion, Moss‐Morris, Silber, Galea, & Chalder, 2010; Jopson & Moss‐Morris, 2003; Spain, Tubridy, Kilpatrick, Adams, & Holmes, 2007; Vaughan, Morrison, & Miller, 2003; Wilski & Tasiemski, 2016a). However, the existing studies provide support for the application of the five‐component structure of illness representations to MS and show that illness representations contribute to MS outcomes such as physical functioning, depression, anxiety and self‐esteem (Vaughan et al., 2003), fatigue (Skerrett & Moss‐Morris, 2006), (social) adjustment (Dennison et al., 2009; Jopson & Moss‐Morris, 2003; Skerrett & Moss‐Morris, 2006), health‐related quality of life (Spain et al., 2007; Wilski & Tasiemski, 2016a), well‐being (Bassi et al., 2016), psychological stress (Dennsion et al., 2010), pain severity and pain interference (Harrison, Silber, McCracken, & Moss‐Morris, 2015), self‐management (Wilski & Tasiemski, 2016b), and—among women—body esteem (Wilski, Tasiemski, & Dąbrowski, 2016). These effects are apparent even when the effects of disease severity (Dennsion et al., 2010; Harrison, Silber et al., 2015; Jopson & Moss‐Morris, 2003; Wilski & Tasiemski, 2016b), physical disability (Spain et al., 2007), or variables such as remission status or mood (Skerrett & Moss‐Morris, 2006) are taken into account. However, as far as we know, no studies have investigated the impact of illness representations on service utilization in MS.

Therefore, the aim of our study was to explore whether, in patients with MS, illness representations are associated with self‐reports of rehabilitation use in the past and the intention to use rehabilitation in the future, beyond sociodemographic and illness‐related factors.

## 2 MATERIALS AND METHODS

### 2.1 Design and participants

We conducted a cross‐sectional nationwide online survey in Germany. The participants (patients over age 18 with MS) were recruited between May and June 2016, predominantly via the website, the Facebook site, and the newsletter of the German Multiple Sclerosis Society (Deutsche Multiple Sklerose Gesellschaft, DMSG), which consists of a federal association, 16 state associations, and about 850 local contact groups. Furthermore, the link to the online survey was distributed via study flyers sent to 27 clinics and specialized medical practices (nationwide) which are listed on the DMSG website and which treat a minimum of 40% patients with MS per year. The study was approved by the Ethics Committee of the University of Freiburg (Approval No. 542/15).

### 2.2 Measures

Illness representations were measured using the German version of the Brief Illness Perception Questionnaire (B‐IPQ; Broadbent, Petrie, Main, & Weinman, 2006). The B‐IPQ is a 9‐item scale designed to rapidly assess the cognitive and emotional representations of illness. The scale measures patients’ cognitive and emotional representations of their illness, including consequences, timeline, personal control, treatment control, identity (symptom burden), coherence, concern, emotional response, and causes. The B‐IPQ items (except the cause item, which uses free text) range from 0 to 10, and a greater score on an item represents a larger value in the measured dimension. According to a systematic review and meta‐analysis, the B‐IPQ is widely used and has good psychometric properties (Broadbent et al., 2015). In our survey, we excluded the cause item and the timeline item, as other studies with MS samples revealed this item to be extremely negatively skewed (Dennsion et al., 2010).

The behavioral variable “use of rehabilitation” in the past (previous attendance) and the intention to use rehabilitation in the future were assessed via the two items “Have you ever made use of rehabilitation because of your MS?” and “Would you make use of rehabilitation because of MS if necessary?” Both items had to be answered dichotomously (“yes” or “no”).

Furthermore, patients completed a questionnaire covering sociodemographic variables (age, sex, education, and family status) and self‐reported information about illness‐related factors (diagnostic subgroup, time since the last exacerbation, time since the first MS symptoms, and time since MS diagnosis).

### 2.3 Data analysis

First, we conducted descriptive analyses to explore the relationships between the included independent variables and the outcome variables: We analyzed the interrelationships between the B‐IPQ items, the relationships between the B‐IPQ items and the sociodemographic variables and illness‐related variables, respectively, and the relationships between all the independent variables and outcome variables (use of rehabilitation and the intention to use rehabilitation) using Pearson correlation coefficients. Correlations of .1 are interpreted as small, correlations ≥.3 as medium, and correlations ≥.5 as strong (Cohen, 1988). Secondly, we applied two separate hierarchical binary logistic regression procedures to analyze whether illness representations are associated with the use of rehabilitation in the past or with the intention to use rehabilitation in the future, over and above sociodemographic and illness‐related variables. In the first step, sociodemographic variables were dummy‐coded and entered into the models. The second step added the dummy‐coded illness‐related variables, and the third step added illness representations. To avoid multicollinearity, the only variables entered during the first and second steps were those that correlated bivariately at *p *<* *.05 with the outcome variables. The following coefficients were interpreted: Nagelkerke *R* ^2^ as an index of the quality of the overall model and the percentage of the variance explained by stages 1–3. Values >.20 can be defined as acceptable, values >.40 are interpreted as good, and values >.50 are interpreted as very good (Backhaus, Erichson, Plinke, & Weiber, 2003). Furthermore, the Wald coefficients and their *p*‐values, odds ratios, and the respective 95% confidence intervals are shown for all included variables of stage 3.

All data analyses were performed using IBM SPSS Statistics 23 (IBM Corp, 2015).

## 3 RESULTS

A total of *N* = 590 patients with MS participated in the study. The vast majority of participants (>90%) were recruited via the DMSG (website, newsletter, or Facebook site), while only 2% of participants were recruited via MS clinics. Women comprised 72.4% of the sample. The mean age was 45.6 years (*SD* = 10.3). Among the patients, 51.7% had relapsing‐remitting MS, and the mean time since the MS diagnosis was 11.0 years (*SD* = 8.5). Two‐thirds of the sample had made use of rehabilitation because of MS in the past and would make use of rehabilitation in the future because of MS if indicated (64.6% and 68.1%, respectively). Table 1 presents the patient characteristics of the sample.

**Table 1:** Sample characteristics: Sociodemographic, illness‐related variables, and outcome variables (use of rehabilitation and intention to use rehabilitation; *N* = 590)

Sociodemographic variables	
Age (*M*,* SD*)	45.6 (10.3)
Sex *N* (%)	
Female	427 (72.4)
Male	152 (25.8)
Living with a partner *N* (%)	
Yes	411 (69.7)
No	171 (29.0)
Level of education *N* (%)	
Elementary school	55 (9.3)
Secondary school	176 (29.8)
Polytechnic secondary school	22 (3.7)
Technical college qualification	102 (17.3)
University qualification	224 (38.0)
Other or no certificate	10 (1.7)

Illness‐related variables	
Diagnostic subgroup	
Relapsing‐remitting MS	305 (51.7)
Primary progressive MS	69 (11.7)
Secondary progressive MS	122 (20.7)
Other	51 (8.6)
Time since the last exacerbation	
<2 months	73 (12.4)
3–6 months	88 (14.9)
7–12 months	83 (14.1)
>12 months	310 (52.5)
Time since the first MS symptoms (*M*,* SD*)	15.7 (9.8)
Time since MS diagnosis (*M*,* SD*)	11.0 (8.5)

Outcome variables	
Use of rehabilitation	
Yes	381 (64.6)
No	209 (35.4)
Intention to use rehabilitation	
Yes	402 (68.1)
No	174 (29.5)

*M* = mean score; *SD* = standard deviation. Totals that do not add up to *N* = 590 are the result of missing values.

The baseline associations between the B‐IPQ items are shown in Table 2. The correlations are predominantly significant, but in the small range. There are medium correlations between personal control and treatment control, concern and identity, concern and emotional response, and consequences and emotional response. Furthermore, the association between identity and consequences is strong.

**Table 2:** Interrelationships between B‐IPQ items (Pearson product‐moment correlation, *p*‐value^a^)

B‐IPQ	Consequences	Personal control	Treatment control	Identity	Concern	Coherence	Emotional response
Consequences	1.0	−.209 (<.001)	−.080 (.056)	**.804 (<.001)**	.237 (<.001)	.107 (.010)	.**301 (<.001)**
Personal control	—	1.0	**.354 (<.001)**	−.190 (<.001)	−.168 (<.001)	.130 (.002)	−.127 (.002)
Treatment control	—	—	1.0	−.014 (.744)	−.014 (.739)	.125 (.003)	−.008 (.841)
Identity	—	—	—	1.0	**.318 (<.001)**	.137 (.001)	.294 (<.001)
Concern	—	—	—	—	1.0	−.110 (.008)	.**592 (<.001)**
Coherence	—	—	—	—	—	1.0	−.062 (.134)

a Correlations >= .3 appear in bold.

Table 3 displays the associations between the B‐IPQ items and the sociodemographic and illness‐related variables. Out of all the 70 correlations, 25 were significant, with *p*‐values <.05. However, with the exception of the correlation between the time since the first symptoms and the perceived consequences (*r *=* *.323), the correlations are all in the small range.

**Table 3:** Relationships between B‐IPQ items and sociodemographic/illness‐related variables (Pearson product‐moment correlation, *p*‐value^a^)

Sociodemographic/illness‐related variables B‐IPQ	Age	Sex	Living with partner	Level of education	Relapsing‐remitting MS	Primary progressive MS	Secondary progressive MS	Time since last exacerbation	Time since first MS symptoms	Time since MS diagnosis
Consequences	.270 (<.001)	−.082 (.048)	.014 (.744)	−.059 (.151)	−.195 (<.001)	.193 (<.001)	.249 (<.001)	.028 (.494)	**.323** (<.001)	.275 (<.001)
Personal control	−.006 (.885)	−.025 (.541)	−.015 (.713)	−.024 (.565)	.028 (.492)	−.058 (.163)	−.063 (.126)	.029 (.485)	.041 (.351)	.056 (.184)
Treatment control	−.075 (.075)	.025 (.545)	−.035 (.395)	−.032 (.437)	.093 (.024)	−.028 (.497)	−.042 (.307)	.032 (.434)	−.024 (.585)	.010 (.819)
Identity	.227 (<.001)	−.102 (.014)	.044 (.287)	−.103 (.013)	−.185 (<.001)	.174 (<.001)	.254 (<.001)	−.031 (.448)	.267 (<.001)	.230 (<.001)
Concern	−.040 (.345)	.023 (.578)	.013 (.744)	.009 (.826)	−.038 (.363)	.059 (.150)	.007 (.867)	−.124 (.003)	−.080 (.066)	−.068 (.106)
Coherence	.068 (.104)	−.028 (.494)	−.069 (.095)	.002 (.969)	.017 (.674)	−.002 (.963)	.126 (.002)	.101 (.014)	.186 (<.001)	.178 (<.001)
Emotional response	−.109 (.010)	.022 (.601)	−.005 (.902)	−.064 (.121)	.065 (.116)	.023 (.582)	−.083 (.044)	−.075 (.069)	−.116 (.008)	−.120 (.004)

a Correlations >= .3 appear in bold; Sex: 1 = female; living with partner: 1 = yes; level of education: 1 = higher (technical college qualification or university qualification).

The results concerning the use of rehabilitation are shown in Table 4. The sociodemographic variables (first step) explained 10.1% of the variance of use of rehabilitation. With the addition of the illness‐related variables (second step), the total explained variance was 15.8%, and with the addition of illness representations (third step), 31.5%. Therefore, the quality of the overall model in the third step was acceptable. In the final model, four variables turned out to be statistically significantly correlated to the use of rehabilitation: not living with a partner, lower level of education, perceived consequences, and—with the highest Wald coefficient—perceived treatment control.

**Table 4:** Prediction of “use of rehabilitation”

Binary logistic regression (*N* = 473)	
First step: Nagelkerke *R* ^2^	10.1
Second step: Nagelkerke *R* ^2^	15.8
Third step: Nagelkerke *R* ^2^	31.5

Variables (third step)	Wald	*p*	Odds ratio	95% confidence interval
Age	1.748	.186	1.018	0.991–1.045
Living with partner^a^	5.551	.018	0.541	0.324–0.902
Level of education^b^	6.601	.010	0.548	0.346–0.867
Diagnostic subgroup: secondary progressive^c^	2.003	.157	1.596	0.835–3.048
Time since last exacerbation	2.356	.125	1.434	0.905–2.274
Time since the first MS symptoms	0.415	.520	1.014	0.972–1.058
Time since MS diagnosis	0.166	.683	1.010	0.963–1.059
Consequences	7.150	.007	1.243	1.060–1.457
Personal control	0.217	.641	0.974	0.870–1.090
Treatment control	18.591	<.001	1.256	1.133–1.394
Identity	3.233	.072	1.164	0.986–1.374
Concern	2.244	.134	0.913	0.810–1.029
Coherence	1.084	.298	1.054	0.955–1.164
Emotional response	0.523	.470	1.043	0.931–1.168

a Living with partner: 1 = yes;

b Level of education: 1 = higher (technical college qualification or university qualification);

c Diagnostic subgroup: secondary progressive: 1 = yes.

Table 5 shows the results with regard to the intention to use rehabilitation. The sociodemographic variables explained 6.2% of the variance. With the addition of the illness‐related variables, the total explained variance was 8.8%, and with the addition of illness representations, it was 25.9%, resulting in an acceptable overall model quality. In the final model, three variables were statistically significantly associated with the intention to use rehabilitation: lower level of education, perceived consequences and—again with the highest Wald coefficient—perceived treatment control.

**Table 5:** Prediction of “intention to use rehabilitation”

Binary logistic regression (*N* = 473)	
First step: Nagelkerke *R* ^2^	6.2
Second step: Nagelkerke *R* ^2^	8.8
Third step: Nagelkerke *R* ^2^	25.9

Variables (third step)	Wald	*p*	Odds ratio	95% confidence interval
Age	2.980	.084	1.024	0.997–1.052
Level of education^a^	7.437	.006	0.521	0.326–0.832
Diagnostic subgroup: secondary progressive^b^	0.150	.699	1.137	0.594–2.174
Time since the first MS symptoms	1.740	.187	1.031	0.985–1.079
Time since MS diagnosis	0.936	.333	0.976	0.928–1.026
Consequences	5.175	.023	1.202	1.026–1.408
Personal control	0.016	.899	0.993	0.886–1.112
Treatment control	24.139	<.001	1.298	1.170–1.440
Identity	2.043	.153	1.124	0.958–1.319
Concern	0.720	.396	1.051	0.937–1.180
Coherence	1.673	.196	1.069	0.966–1.182
Emotional response	0.002	.969	1.002	0.897–1.120

a Level of education: 1 = higher (technical college qualification or university qualification).

b Diagnostic subgroup: secondary progressive: 1 = yes.

The same procedure also controlling for use of rehabilitation in the past (in the first step) showed that perceived past behavior explained 56% of the variance of intention to use rehabilitation. The explained variance was slightly higher when including the sociodemographic and illness‐related variables (+ 1.5%) and including illness representations yielded a further increase in the explained variance, by four percentage points. Besides use of rehabilitation, with the highest Wald coefficient (Wald = 115.099; *p *<* *.001), treatment control (Wald = 7.914; *p *=* *.005) and concern (Wald = 6.808; *p *=* *.009) were also significantly associated with the intention to use rehabilitation.

## 4 DISCUSSION

The results of our study show that certain illness representations are related to the intention to use and the use of rehabilitation in MS, above and beyond sociodemographic and illness‐related factors: The probabilities of intention to use rehabilitation and of use of rehabilitation were higher in patients who believed that their MS was controllable by treatment and perceived that their MS would have severe consequences. Illness representations explained a higher proportion of variance than sociodemographic and illness‐related variables, and the illness representation domain “treatment control” showed the strongest relationship within both regression models. The relevance of illness representations was further underlined in the third regression analysis, where perceived past behavior (rehabilitation use) was controlled for the intention‐related outcome variable.

With respect to the outcome domain, our results are largely in line with studies in the context of cardiac rehabilitation. The illness representation domains “cure/control,” “consequences,” “identity,” and “coherence” were significantly associated with attendance at cardiac rehabilitation in patients with acute myocardial infarction (French et al., 2006), and they also turned out to be predictors of dropout from a cardiac rehabilitation program (Yohannes et al., 2007).

With respect to the diagnostic group of patients with MS, our results are consistent with Vaughan et al. (2003), who reported plausible correlations between some illness representation components, mainly in the small to medium range. However, the results are not fully comparable because of the different kinds of operationalization of the illness representations (Vaughan et al. (2003) used the Illness Perception Questionnaire (Weinman, Petrie, Moss‐Morris & Horne, 1996) before its revision, while the B‐IPQ is based on the revised version of the Illness Perception Questionnaire (Moss‐Morris et al., 2001)).

In our models, a lower level of education correlated with both outcome variables, while not living with a partner was correlated with the use of rehabilitation. We are not aware of any studies investigating the impact of sociodemographic variables on rehabilitation use in MS. However, in other illness groups, such as myocardial infarction, results of a systematic review demonstrate that demographic variables such as age, sex, employment status, education, and income clearly affect participation in and adherence to cardiac rehabilitation programs (Ruano‐Ravina et al., 2016). Focusing on social roles and patients’ life contexts, which are related to sociodemographic variables to some degree, Helland et al. (2015) showed, in their qualitative study in patients with MS, that practical barriers in a patient's work or family life, such as caring for small children or other family members, prohibited a rehabilitation stay. However, in view of the small amount of empirical data, it seems worthwhile to pay more attention to sociodemographic variables and their influence on service utilization in MS in further studies.

With respect to the illness‐related variables, none of the variables proved to be significantly correlated with the outcomes. On the one hand, this can be seen as being in line with studies which show that illness‐related factors are not consistently associated with adjustment outcomes in MS and often only predict modest amounts of the variance (Dennsion et al., 2010). On the other hand, the lack of influence of illness‐related variables may be due to the fact that we used self‐reported measures to assess these variables. However, illness‐related variables are often determined using self‐reported measures (Dennsion et al., 2010; Harrison, Silber et al., 2015; Jopson & Moss‐Morris, 2003) and—as a review of the psychological correlates of adjustment in patients with MS shows—many studies completely fail to report important medical characteristics about the participants, including MS type, disease severity, and time since diagnosis (Dennison et al., 2009). Taking into account these considerations, we consider the self‐reported measures as reasonable proxies for the measurement of the illness‐related variables in our study. However, including a physician‐based measurement of illness‐related variables should be considered as a good way to complement our study design.

Furthermore, our models showed that certain illness representations, namely perceived consequences and treatment control, have the strongest relationship with the use and the intention to use rehabilitation in MS. The consequences domain seems to be associated with many outcomes in MS, such as illness intrusiveness, physical functioning, depression, self‐esteem, anxiety (Vaughan et al., 2003), health‐related quality of life (Spain et al., 2007), pain severity, and pain interference (Harrison, Silber et al., 2015). Furthermore, the remaining illness representation domains have also been shown to be associated with important outcomes. For example, the identity domain has been identified as associated with the quality of life (Spain et al., 2007; Wilski & Tasiemski, 2016a) and functional impairment (Dennsion et al., 2010), illness coherence with psychological stress (Dennsion et al., 2010), the perceived timeline with pain‐related outcomes (Harrison, Silber et al., 2015), and treatment control with self‐management (Wilski & Tasiemski, 2016b). Nevertheless, these results do not allow a conclusion about which of the illness representation domains are the most important for a specific outcome. On the one hand, the above‐mentioned studies indeed share some similarities—most of them used the IPQ‐R (Moss‐Morris et al., 2001) or B‐IPQ to assess illness representations, and many of them applied hierarchical (linear) regression analyses, where demographic and disease factors were entered in the first step and psychosocial factors in the second step. On the other hand, there are important differences regarding the included samples, included illness representation domains, and demographic and illness‐related variables, suggesting that a range of illness representations might be important in explaining individual differences in adjustment outcomes.

As far as we know, no studies have investigated the impact of illness representations on service utilization in MS. Nor are we aware of any studies focusing on the utility of illness representations within the theoretical framework of the CSM in the context of the adherence literature in MS, which could be seen as a construct related to service utilization. However, on a superordinate construct level, evidence suggests that adherence to disease‐modifying therapies (DMT) is influenced by patients’ attitudes and (adherence) expectations, treatment beliefs such as the perceived benefits of DMT therapy, and self‐efficacy (Brandes, Callender, Lathi, & O'Leary, 2009; Jongen, Lemmens, Hoogervorst, & Donders, 2017; Turner, Kivlahan, Sloan, & Haselkorn, 2007; Turner, Roubinov, Atkins, & Haselkorn, 2016; Zwibel, Pardo, Smith, Denney, & Oleen‐Burkey, 2011).

In sum, the evidence of the relevance of illness representations for different outcomes justifies their further consideration in the clinical context and in future research. From a clinical point of view, illness representations are to be considered as modifiable variables and, therefore, are promising targets for patient‐oriented psychological interventions to support patients’ adjustment (Dennsion et al., 2010; Harrison, McCracken, Bogosian, & Moss‐Morris, 2015; Harrison, Silber et al., 2015; Jopson & Moss‐Morris, 2003; Skerrett & Moss‐Morris, 2006; Spain et al., 2007; Vaughan et al., 2003; Wilski & Tasiemski, 2016a, 2016b). As a result of their review of the psychological correlates of adjustment in patients with MS, Dennison et al. (2009) made concrete suggestions with respect to the content and the delivery of psychological interventions embedded in a cognitive–behavioral model. Within this framework, illness (and treatment) representations might reflect illness‐specific and patient‐specific intervention targets which could be addressed in an educational setting. In the context of medical rehabilitation in Germany, many patient education programs aiming at patients’ empowerment and self‐management have been developed and evaluated in the last decade. These programs include illness and treatment‐related information, skills training (i.e., self‐management skills), motivation to establish a healthy lifestyle, stress management, and psychological elements (Bitzer et al., 2009) and, therefore, present a good opportunity for the explicit inclusion of illness representation interventions. In patients with chronic back pain, the delivery of such an intervention in a rehabilitation context was seen as promising (Glattacker, Heyduck, & Meffert, 2012). With the aim of facilitating the *intention* to use rehabilitation, the delivery of such interventions in other settings is required. However, as perceived treatment control seems to play a predominant role as a facilitator of utilization, such interventions should focus on fostering patients’ treatment expectations—for example, through the provision of comprehensive information about the processes and aims of multidisciplinary rehabilitation approaches. However, illness and treatment representations are constantly modified in a broad personal, social, and cultural context (Leventhal et al., 2001), resulting in the fact that illness and treatment representations can show considerable intra‐ and interindividual variability. Therefore, the context of the development of illness and treatment representations such as the individual's illness history or social interaction with the family or professionals should be taken into account when implementing such interventions.

Focusing on the quality of research on illness representations, the limitations pointed out in the review of Dennison et al. (2009) are still valid in more recent papers: Most studies are cross‐sectional (Dennsion et al., 2010; Harrison, Silber et al., 2015; Wilski & Tasiemski, 2016a, 2016b), sample sizes are often small (below 150), and the independent and outcome variables are predominantly measured via self‐report questionnaires (Dennison et al., 2009). Future research would benefit from studies without these limitations, using longitudinal designs and large samples. Furthermore, in order to guide the development of psychological interventions, it would be useful to investigate the relative importance of different—often overlapping—psychological variables and their roles as mediator or moderator variables (Dennison et al., 2009) in a theory‐driven way.

A major limitation of our study is its cross‐sectional design. Furthermore, all variables—including diagnostic subgroup—were measured using self‐report questionnaires. This implies the potential of a recall bias, for example with respect to our retrospective assessed behavioral outcome variable “rehabilitation use”. Operationalizing the intention to use rehabilitation in a binary way might have been too simplistic. Furthermore, it must be taken into account that an intention does not necessarily predict behavior such as participation in rehabilitation. The recruitment strategy implies the potential for selection bias: No information is available about people with MS who declined to take part in this online study. However, the sample was large and highly comparable with respect to (German) epidemiological data found in the literature (Stuke et al., 2009). Another limitation lies in the fact that we refrained from measuring other independent variables that could have been relevant, such as MS severity or mood—although there is preliminary evidence which shows that illness representations are not just a reflection of mood states in MS (Harrison, Silber et al., 2015; Skerrett & Moss‐Morris, 2006). Finally, we excluded the illness representation domain timeline due to the negative skewness in other MS samples (Dennsion et al., 2010) and excluded the cause item due to its open format, which would have involved a different issue and separate analyses (see Bassi et al., 2016).

## 5 CONCLUSION

Illness representations are related to the intention to use and the use of rehabilitation in MS, above and beyond sociodemographic and illness‐related factors. Although, as far as we know, this study is the first of its kind in a German MS population to include a large sample and control for demographic and illness‐related variables, the results should be replicated in longitudinal studies to overcome the above‐mentioned limitations. However, our data suggest that addressing patients’ illness representations may facilitate the intention to use and the use of multimodal rehabilitation, which is recommended as an important treatment option in MS, one that contributes to better illness outcomes.

## CONFLICT OF INTEREST

Angelika Nebe works at the German Pension Insurance (see affiliation). The authors have no further competing interests to report.
